# The Effect of Diabetic Neuropathy on Recurrent Laryngeal Nerve Monitoring During Thyroid Surgery

**DOI:** 10.7759/cureus.94739

**Published:** 2025-10-16

**Authors:** Eustratios Kofopoulos-Lymperis, Dimitrios Papakonstantinou, Konstantinos Chaidas, Alexander Delides, Matthew Stamelos, Danae Kakabakou, Emmanouil Pikoulis, Evangelos Missiakos, Melpomeni Peppa, Konstantinos Nastos

**Affiliations:** 1 Third Department of Surgery, "Attikon" University General Hospital/National and Kapodistrian University of Athens, School of Medicine, Athens, GRC; 2 Department of Ear, Nose, and Throat, University Hospital of Alexandroupolis, Democritus University of Thrace, School of Medicine, Alexandroupolis, GRC; 3 Second Department of Otolaryngology, "Attikon" University General Hospital/National and Kapodistrian University of Athens, School of Medicine, Athens, GRC; 4 Department of Surgery, Evgenidion Hospital, Athens, GRC; 5 Second Department of Propaedeutic Internal Medicine, "Attikon" University General Hospital/National and Kapodistrian University of Athens, School of Medicine, Athens, GRC

**Keywords:** amplitude, intraoperative neuromonitoring (ionm), latency, recurrent laryngeal nerve (rln), thyroid surgery, types 2 diabetes

## Abstract

Background: Recurrent laryngeal nerve (RLN) injury remains a significant complication of thyroid surgery, potentially leading to vocal cord paresis and compromised quality of life. Intraoperative nerve monitoring (IONM) enables real-time assessment of RLN function but may be affected by underlying neuropathies. Diabetes mellitus (DM) induces peripheral nerve damage, potentially altering RLN electrophysiology during surgery. This study investigates the impact of DM on RLN amplitude and latency during thyroidectomy.

Methods: We prospectively analyzed 245 patients (485 RLNs at risk) who underwent thyroidectomy with IONM between 2020 and 2024. Patients were classified as diabetic or non-diabetic according to American Diabetes Association (ADA) criteria. Standardized surgical and anesthetic protocols were followed. RLN amplitude and latency were measured before and after dissection using consistent stimulation parameters, and group comparisons were performed using non-parametric tests.

Results: Thirty-four patients (13.9%) had diabetes, representing a more aged cohort with a higher prevalence of comorbidities than non-diabetic patients. Pre-dissection RLN amplitude and latency did not differ significantly between groups (0.72 mV vs. 0.74 mV, p = 0.87; 2.2 ms vs. 1.9 ms, p = 0.11, respectively). After dissection, diabetic patients showed a significantly greater reduction in RLN amplitude (median 0.58 mV vs. 0.77 mV in non-diabetics, p = 0.01), whereas latency remained comparable (p = 0.92). Within-group analysis confirmed a significant amplitude drop among diabetics (p = 0.008) but not among non-diabetics. Temporary unilateral vocal fold palsy occurred in three patients (one diabetic, two non-diabetic), all resolving within two weeks.

Conclusions: Diabetic patients demonstrated greater RLN amplitude reduction during thyroidectomy, suggesting impaired neural resilience to surgical manipulation. However, these electrophysiological changes had minimal short-term clinical impact when nerve integrity was preserved. Awareness of this subclinical vulnerability may guide intraoperative decision-making within the context of neuroprotective strategies that involve IONM during thyroidectomy.

## Introduction

Recurrent laryngeal nerve (RLN) injury is a major complication of thyroid surgery, occasionally causing temporary or permanent vocal cord paresis, significantly impairing patients' quality of life due to hoarseness or aspiration. While intraoperative visual identification of the RLN has traditionally been used to prevent injury, anatomical integrity does not guarantee functional preservation. To address this, intermittent intraoperative nerve monitoring (IONM) has been developed and implemented in thyroid surgery, enabling real-time assessment of RLN functionality [1].

Diabetes mellitus (DM) is known to induce degeneration of myelin and axonal structures in peripheral nerves [2], leading to diabetic peripheral neuropathy (DPN). In the U.S., approximately 3.9% of the population has DM, and 50% of these patients develop some degree of DPN. DPN affects the central, peripheral, somatic, and autonomic nervous systems through multifactorial mechanisms. Symmetrical diabetic polyneuropathy typically manifests distally, involving both myelinated and unmyelinated fibers and causing axonal degeneration in sensory, motor, and autonomic neurons [3]. Electrophysiological studies reveal reduced nerve potential amplitudes and prolonged conduction times, with magnitude changes that depend on the severity and type of nerve damage [4]. 

During thyroidectomy, the RLN is at risk of transection, ligation, thermal, or traction injuries, any of which may result in postoperative vocal cord paresis [5]. The introduction of IONM aims to reduce the occurrence of such complications by monitoring for reductions in the amplitude of nerve excitation potentials along with prolongations in nerve conduction times (latency) [6]. However, the impact of pre-existing diabetic neuropathy on RLN functional integrity during surgery remains unclear. This study aims to determine whether DM affects RLN amplitude and conduction time during intraoperative stimulation in thyroidectomy patients.

## Materials and methods

Study population

Adult patients undergoing thyroid surgery, for both benign and malignant lesions, with concurrent use of IONM at the Attikon University Hospital and Eugenideio Hospital, between January 2020 and January 2024 were prospectively evaluated for inclusion in this study. Patients were excluded from the study in cases of pre-existing vocal cord paresis, as documented by routine preoperative laryngoscopy. Cases in which intraoperative visual identification of the RLN was not possible or in which IONM did not function due to technical reasons were also excluded. Written informed consent is obtained from all participating patients, and the study was approved by the local institutional ethics committees (ID number: 191/06-03-2024).

For the purposes of this study, patients were divided into two groups: (1) Diabetic patients: Those meeting the diagnostic criteria for DM according to the American Diabetes Association (ADA) [7] and receiving antidiabetic medication and (2) Non-diabetic patients: Those not meeting ADA criteria for DM [7].

Anesthesia management

During anesthesia induction, either 2-2.5 mg/kg succinylcholine or small doses of non-depolarizing muscle relaxants were administered to facilitate rapid restoration of vocal cord muscle activity during thyroid dissection. The recording of neurostimulation signals was performed only after the effects of any muscle relaxants had been fully reversed [8].

To ensure accurate documentation of muscle responses, an endotracheal tube with an integrated electrode was used. The electrode was positioned 7-10 mm above the upper limit of the cuff, ensuring consistent placement at the level of the glottis and contact with the inner surface of both vocal cords. The tube was stabilized after positioning the head in full extension to prevent electrode displacement during surgery [8].

Surgical approach

All procedures were performed by the same high-volume endocrine surgeon (with a yearly caseload of more than 100 thyroidectomies) using a standardized anesthetic and surgical technique. During thyroidectomy, the RLN was identified in the tracheoesophageal groove after releasing the upper pole of the thyroid and before its crossing with the branches of the inferior thyroid artery. The RLN was initially stimulated immediately caudally to the crossing point with the inferior thyroid artery, per protocol. The branches of the inferior thyroid artery were then identified and ligated. The RLN was carefully dissected from surrounding fatty tissue and traced to Berry’s ligament. After dissecting the thyroid lobe from Berry’s ligament, the RLN was stimulated again at the same point as initially done, to record post-thyroidectomy neurostimulation values.

Neuromonitoring parameters

Neurostimulation parameters were defined based on the electromyographic waveform of the vocal cord muscles during bipolar stimulation.

Amplitude

The biphasic waveform represents the sum of motor potentials from the ipsilateral vocal cord muscles. Amplitude correlates with the number of muscle fibers depolarized during stimulation, with normal phonation values ranging between 100 and 800 μV [6]*.*

Latency

This parameter reflects the time from stimulation to the onset of the waveform, recorded in milliseconds (ms). Latency is influenced by the distance between the stimulation site and the ipsilateral vocal cord [6].

The position of the RLN was initially mapped using a 3 mA stimulus, after which all amplitude and latency measurements were performed using a consistent 1.5 mA stimulus. Measurements included an initial pre-thyroidectomy stimulation of the RLN at the level of the crossing of the RLN with the inferior thyroid artery and repeated post-thyroidectomy at the same location to ensure reproducibility. Immediate postoperative laryngoscopy was employed routinely to document vocal fold functionality.

For intraoperative neuromonitoring, the Avalanche SI Neuromonitor (Dr. Langer Medical GmbH, Waldkirch, Germany) was used and it was performed in accordance with international standards [6].

Statistical analysis

Descriptive statistics were used to summarize baseline patient demographics and neurostimulation measurements using IBM SPSS Statistics for Windows, Version 27 (Released 2020; IBM Corp., Armonk, New York, United States). The normality of continuous data was assessed using the Kolmogorov-Smirnov test. Due to the non-normal distribution of the data, continuous variables were reported as median and range. Categorical baseline variables were compared between diabetic and non-diabetic groups using Fisher’s exact test.

Amplitude and latency values were compared between diabetic and non-diabetic patients during the pre- and post-dissection phases using the non-parametric Mann-Whitney U test. Paired comparisons of pre- and post-dissection values within each group were performed using the Wilcoxon signed-rank test. A p-value threshold of 0.05 was used to determine statistical significance.

## Results

Baseline patient characteristics

A total of 245 patients, with 485 RLNs at risk, were included in the study, of which 34 (13.9%) were diabetic patients and 211 (86.1%) were non-diabetic patients. Diabetic patients were significantly older, with a median age of 57 years (range: 18-80) compared to 49.5 years (range: 18-87) in non-diabetic patients (Table 1, p < 0.001). There was baseline equivalence in gender distribution between the two groups (p = 0.33), with female patients representing 55.9% of diabetic patients and 65.9% of non-diabetic patients. Comorbidities such as smoking (70.6% vs. 21.8%, p < 0.001), hypertension (58.8% vs. 23.2%, p < 0.001), and dyslipidemia (73.5% vs. 39.8%, p < 0.001) were significantly more prevalent in diabetic patients. The primary indication for thyroidectomy also differed between groups, with malignancy being more common in diabetic patients (79.4% vs. 54.5%, p = 0.001), while thyrotoxicosis was exclusively observed in non-diabetic patients (20.9%). Regarding the extent of surgery, the number of patients undergoing total thyroidectomy or lobectomy alone and that undergoing thyroidectomy with lateral lymph node dissection were comparable between groups (p > 0.05). However, central lymph node dissection was less frequently performed in diabetic patients (17.6% vs. 51.2%, p < 0.001).

**Table 1:** Baseline patient demographics. ^a  ^Mann Whitney U test ^b ^Fisher's Exact Test *Values are presented as median (range).

	Total n, (%)	Diabetic patients n, (%)	Non-diabetic patients n, (%)	p-value
Age*		57 (18-80)	49.5 (18-87)	<0.001^a^ (Mann Whitney-U=6074)
Gender				0.33^b^
Female	158	19 (55.9)	139 (65.9)	
Male	87	15 (44.1)	72 (34.1)	
Comorbidities				0.25^b^
Smoking	70	24 (70.6)	46 (21.8)	
Hypertension	69	20 (58.8)	49 (23.2)	
Dyslipidemia	109	25 (73.5)	84 (39.8)	
Indication for resection				0.001^b^
Malignancy	142	27 (79.4)	115 (54.5)	
Compression	58	7 (20.6)	51 (24.2)	
Thyrotoxicosis	44	0	44 (20.9)	
Operation				0.09^b^
Lobectomy	5	0	5 (2.4)	
Thyroidectomy	240	34 (100)	206 (97.6)	
Central lymph node dissection	114	6 (17.6)	108 (51.2)	
Lateral lymph node dissection	36	4 (11.8)	32 (15.2)	

Perioperative neurophysiological changes

Neurostimulation parameters of the RLN between diabetic and non-diabetic patients were compared in both the pre-dissection and post-dissection phases of surgery (Table 2). Before dissection, there were no significant differences in RLN amplitude (0.72 mV vs. 0.74 mV, p = 0.87) or latency (2.2 ms vs. 1.9 ms, p = 0.11) between the two groups. However, post-dissection, diabetic patients exhibited significantly reduced median RLN amplitude values compared to non-diabetic patients (0.58 mV vs. 0.77 mV, p = 0.01, Table 2), while latency remained comparable between the two groups (p = 0.92).

**Table 2:** Characteristics of the perioperative changes in recurrent laryngeal nerve amplitude and latency in diabetic and non-diabetic patients. ^a ^Mann Whitney U Test ^b ^Wilcoxon signed-rank test All values are presented as median (range).

	Before-dissection					After-dissection			
	Diabetic	Non-diabetic	Mann-Whiteny U test statistic	p-value^a^		Diabetic	Non-diabetic	Mann-Whiteny U test statistic	p-value^a^
Amplitude (mV)	0.72 (0.15-1.63)	0.74 (0.09-3.2)	3587	0.87		0.58 (0.13-1.61)	0.77 (0.07-2.7)	2599	0.01
Latency (ms)	2.2 (1-3.9)	1.9 (1-39)	3940	0.11		1.95 (1-3.8)	2 (0.9-4)	3621	0.92
	Diabetic patients					Non-Diabetic patients			
	Before-dissection	After-dissection	Wilcoxon signed-rank test W statistic		p-value^b^	Before dissection	After-Dissection	Wilcoxon signed-rank test W statistic	p-value^b^
Amplitude (mV)	0.72 (0.15-1.63)	0.58 (0.13-1.61)	6		0.008	0.74 (0.09-3.2)	0.77 (0.07-2.7)	9	0.01
Latency (ms)	2.2 (1-3.9)	1.95 (1-3.8)	59		0.22	1.9 (1-39)	2 (0.9-4)	43	0.16

Intragroup analysis revealed a significant drop in RLN amplitude in diabetic patients following dissection (0.72 mV to 0.58 mV, p = 0.008), whereas non-diabetic patients showed a significant increase in amplitude values (0.74 mV to 0.77 mV, p = 0.01). Latency changes were not significant in either group.

The percentage perioperative change in RLN amplitude was also more pronounced in diabetic patients (-1% vs. 6.9%, p = 0.11), though this difference did not reach statistical significance. Similarly, the percentage change in latency was greater in diabetic patients (-3.6% vs. 0%, p = 0.056), approaching but not achieving statistical significance.

Three cases of postoperative unilateral vocal fold palsy (VFP) (two in the non-diabetic group and one in the diabetic group) were identified and confirmed using laryngoscopy. Complete remission was observed within two weeks of follow-up.

Before dissection, there were no significant differences in RLN amplitude (0.72 mV vs. 0.74 mV, p = 0.87) or latency (2.2 ms vs. 1.9 ms, p = 0.11) between the two groups. However, post-dissection, diabetic patients exhibited significantly reduced median RLN amplitude values compared to non-diabetic patients (0.58 mV vs. 0.77 mV, p = 0.01, Table 2), while latency remained comparable between the two groups (p= 0.92). Intragroup analysis revealed a significant drop in RLN amplitude in diabetic patients following dissection (0.72 mV to 0.58 mV, p = 0.008), whereas non-diabetic patients showed a significant increase in amplitude values (0.74 mV to 0.77 mV, p = 0.01). Latency changes were not significant in either group. The percentage perioperative change in RLN amplitude was also more pronounced in diabetic patients (-1% vs. 6.9%, p = 0.11), though this difference did not reach statistical significance. Similarly, the percentage change in latency was greater in diabetic patients (-3.6% vs. 0%, p = 0.056), approaching but not achieving statistical significance.

Three cases of postoperative unilateral VFP (two in the non-diabetic group and one in the diabetic group) were identified and confirmed using laryngoscopy. In the two cases of non-diabetic patients, a perioperative amplitude drop on the side of the affected nerve from 1.52 mV to 0.77 mV was noted in the first case and a change from 0.91 mV to 0.66 mV was registered in the second case. Attained perioperative latency readings from the same nerves were 1.8 ms and 1.4 ms preoperatively and 1.9 ms and 1.6 ms postoperatively. Regarding the diabetic patient that demonstrated postoperative VFP, preoperative amplitude and latency were 0.39 mV and 2.1 ms, which after dissection changed to 0.27 mV and 2.4 ms, respectively. In all three cases, nerves were found to be anatomically intact. Complete remission was observed within two weeks of follow-up.

## Discussion

This study aims to determine whether pre-existing diabetic neuropathy alters RLN electrophysiology during thyroidectomy, as measured by IONM. Our findings demonstrate that while diabetic and non-diabetic patients exhibit comparable baseline RLN amplitudes and latencies, diabetics show a significant post-dissection amplitude reduction (0.72 mV to 0.58 mV, p = 0.008), a pattern absent in non-diabetics. These results suggest that diabetic nerves may be less resilient to surgical manipulation, even when anatomical integrity is preserved. This observation aligns with broader evidence that DM impacts peripheral nerve function, but contrasts with reports presenting conflicting evidence about its clinical significance in thyroid surgery.

In the present patient cohort, RLNs of diabetic patients were more prone to amplitude reductions, while latency remained unchanged and postoperative VFP was rare and reversible. The amplitude decline in diabetics may reflect reduced neural reserve or impaired capacity for rapid functional recovery following manipulation, consistent with the broader pathophysiology of diabetic neuropathy. Nevertheless, the clinical impact of such alterations is, in most cases, negligible provided that the anatomic integrity of the RLN is preserved. The paradoxical increase in amplitude observed in non-diabetic patients indicates that nerve responses are divergent, emphasizing that preexisting neuropathy may lead to different nerve conduction responses to surgical handling.

From a surgical standpoint, these findings suggest that the presence of diabetes does not need to alter the standard surgical approach. Intraoperative neuromonitoring still acts as a potential safeguard; however, subtle conduction changes should not be overinterpreted as predictors of permanent dysfunction but rather as indicators of RLN transient stress with limited vocal cord function impact.

Older studies report a higher incidence of surgery-independent vocal cord palsy in diabetic patients compared to non-diabetics [9], with both unilateral and bilateral paralysis linked to DM [10]. Electrophysiological studies further suggest that diabetic neuropathy may increase RLN susceptibility to injury [11,12], implying that the vagus and recurrent laryngeal nerves could be affected similarly to peripheral nerves. Laryngeal sensory neuropathy (LSN), caused by superior or recurrent laryngeal nerve dysfunction, manifests as throat discomfort (for example, paresthesia, pain), chronic dysphonia, laryngospasm, or cough [13-15]. A 2014 cross-sectional study by Hamdan et al. [16] found LSN in 42% of type 2 diabetic patients (n=50) versus 13.9% of controls (n=36), highlighting its clinical relevance in diabetes.

Similarly, Schlosser et al. [17] retrospectively analyzed 630 thyroidectomy patients and found comparable rates of perioperative VFP between diabetics (11.4%) and non-diabetics (8.4%), though permanent VFP was marginally higher in diabetics (4.5% vs. 1.4%). In contrast, Chen et al. [18] identified diabetes as a risk factor for iatrogenic VFP (2.1% incidence in 2,815 patients), particularly in those >60 years old or undergoing total thyroidectomy with neck dissection.

Another retrospective cohort study by Chung et al. [19] with patients recruited from 2002 to 2013, analyzed the thyroidectomy complications in patients with diabetes mellitus. This retrospective multi-institutional analysis used the NIS database with a sample size of. 100,000 cases and did not find correlation between diabetic status and vocal cord paresis/paralysis following thyroid surgery, although the variant analysis showed a significantly higher rate of vocal cord paralysis in the DM cohort after thyroid surgery. This significance was no longer apparent in the multivariate analysis that controlled for demographic factors and comorbidities.

Serpell et al. [20] investigated intraoperative changes in RLN morphology during thyroidectomy, demonstrating a significant increase in nerve diameter from 1.95 mm to 2.64 mm (mean difference: 0.71 mm; p < 0.001) in their study of 110 RLNs from 75 patients. This morphological change was accompanied by a paradoxical increase in EMG amplitude from 493.5 μV to 594.5 μV (mean difference: 101 μV). The authors attribute temporary postoperative RLN palsy primarily to neurapraxia, a transient conduction block classified under Seddon's nerve injury spectrum (alongside axonotmesis and neurotmesis) resulting from intraoperative mechanical stress (stretching, compression, or manipulation) [21].

While nerve edema theoretically predisposes to neurapraxia by increasing diameter, the observed amplitude elevation suggests alternative physiological mechanisms. The authors hypothesize that edema may modify ionic channel function, potentially enhancing nerve excitability. This counterintuitive finding highlights the complex relationship between RLN morphology and electrophysiology during thyroid surgery, though the precise etiology of this edema-mediated excitability remains unclear. This is further compounded by the potential effect of muscle-relaxing agents whose pharmacodynamics may exhibit interpatient variability.

Building upon their earlier findings, Serpell et al. [22] conducted a larger-scale analysis of 3,408 thyroidectomies (5,334 nerves at risk) to evaluate laterality differences in RLN palsy rates. They documented postoperative palsy in 82 patients (1.3% temporary, 0.3% permanent), with distinct anatomical patterns: right-sided nerves showed higher vulnerability during bilateral procedures, while left-sided nerves were more frequently affected in unilateral cases. The study confirmed their previous observations of intraoperative RLN swelling, with mean diameter increasing from 1.6 mm to 2.4 mm. Electrophysiological changes differed by side, with left RLNs demonstrating greater amplitude elevation (634 μV to 747 μV) compared to right RLNs (548 μV to 575 μV). These findings suggest that anatomical and surgical factors may interact differently with left versus right RLNs, potentially explaining the laterality-specific palsy patterns observed. In the present study, the observed increase in amplitude in the non-diabetic group, although statistically significant, is minimal in terms of magnitude and likely in terms of clinical significance as well.

Several limitations of this study warrant consideration. First, while our cohort included a substantial number of patients, the relatively small proportion of diabetic participants (n=34) may limit the generalizability of our findings. Moreover, although several imbalances were noted in patient baseline demographics due to non-matching, the small sample size precluded the use of multivariable analysis, Second, the study design did not include long-term follow-up to assess whether the observed electrophysiological changes correlated with any clinically evident voice impairment. Third, we could not account for potential confounding factors such as varying durations and severity of diabetes, glycemic control, or the presence of other microvascular complications. In addition, diabetic patients in our cohort were significantly older and had a higher burden of comorbidities, which may themselves influence RLN electrophysiology and contribute to the observed differences in amplitude. Fourth, all procedures were performed by a single high-volume surgeon, which may affect the reproducibility of results in different surgical settings. Finally, while intraoperative neuromonitoring provides valuable real-time data, the technique has inherent limitations (such as the variability in produced responses, depending on the exact point of the nerve that is stimulated) in assessing subtle neural changes that may not manifest in amplitude or latency measurements. These constraints highlight the need for broader, multicenter studies with standardized diabetic classification and neuromonitoring practices to validate our observations, particularly since no cases of permanent vocal cord palsy were observed in our cohort, limiting the direct assessment of long-term clinical relevance.

## Conclusions

In conclusion, this study demonstrates that diabetic patients exhibit significant post-dissection reductions in RLN amplitude during thyroidectomy, despite comparable baseline electrophysiology to non-diabetics. While the clinical implications of these findings require further investigation, they suggest that pre-existing diabetic neuropathy may compromise RLN resilience to surgical manipulation. The paradoxical amplitude increases observed in non-diabetics highlight the complexity of intraoperative nerve physiology. Future research should correlate these electrophysiological changes with long-term vocal outcomes and explore targeted neuroprotective strategies for diabetic patients undergoing thyroid surgery.

## References

[1] Schneider R, Randolph GW, Dionigi G (2018). International neural monitoring study group guideline 2018 part I: staging bilateral thyroid surgery with monitoring loss of signal. Laryngoscope.

[2] Feldman EL, Callaghan BC, Pop-Busui R (2019). Diabetic neuropathy. Nat Rev Dis Primers.

[3] Chong ZZ, Menkes DL, Souayah N (2024). Targeting neuroinflammation in distal symmetrical polyneuropathy in diabetes. Drug Discov Today.

[4] Shabeeb D, Najafi M, Hasanzadeh G, Hadian MR, Musa AE, Shirazi A (2018). Electrophysiological measurements of diabetic peripheral neuropathy: a systematic review. Diabetes Metab Syndr.

[5] Dionigi G, Wu CW, Kim HY, Rausei S, Boni L, Chiang FY (2016). Severity of recurrent laryngeal nerve injuries in thyroid surgery. World J Surg.

[6] Randolph GW, Dralle H, Abdullah H (2011). Electrophysiologic recurrent laryngeal nerve monitoring during thyroid and parathyroid surgery: international standards guideline statement. Laryngoscope.

[7] (2019). 2. Classification and diagnosis of diabetes: standards of medical care in diabetes-2019. Diabetes Care.

[8] Pang G, Edwards MJ, Greenland KB (2010). Vocal cords-carina distance in anaesthetised Caucasian adults and its clinical implications for tracheal intubation. Anaesth Intensive Care.

[9] Berry H, Blair RL (1980). Isolated vagus nerve palsy and vagal mononeuritis. Arch Otolaryngol.

[10] Kabadi UM (1988). Unilateral vocal cord palsy in a diabetic patient. Postgrad Med.

[11] Polednak AP (2009). Vocal fold palsy after surgery in elderly thyroid cancer patients with versus without comorbid diabetes. Surgery.

[12] Ozemir IA, Ozyalvac F, Yildiz G, Eren T, Aydin-Ozemir Z, Alimoglu O (2016). Importance of latency and amplitude values of recurrent laryngeal nerve during thyroidectomy in diabetic patients. Int J Surg.

[13] Amin MR, Koufman JA (2001). Vagal neuropathy after upper respiratory infection: a viral etiology?. Am J Otolaryngol.

[14] Bastian RW, Vaidya AM, Delsupehe KG (2006). Sensory neuropathic cough: a common and treatable cause of chronic cough. Otolaryngol Head Neck Surg.

[15] Lee JK, Mintz S (2006). Chronic cough as a sign of laryngeal sensory neuropathy: diagnosis and treatment. Ann Otol Rhinol Laryngol.

[16] Hamdan AL, Dowli A, Barazi R, Jabbour J, Azar S (2014). Laryngeal sensory neuropathy in patients with diabetes mellitus. J Laryngol Otol.

[17] Schlosser K, Maschuw K, Hassan I (2008). Are diabetic patients at a greater risk to develop a vocal fold palsy during thyroid surgery than nondiabetic patients?. Surgery.

[18] Chen HC, Pei YC, Fang TJ (2019). Risk factors for thyroid surgery-related unilateral vocal fold paralysis. Laryngoscope.

[19] Chung SY, Govindan A, Babu A, Tassler A (2019). Thyroidectomy complications in patients with diabetes mellitus. Otolaryngol Head Neck Surg.

[20] Serpell JW, Woodruff S, Bailey M, Grodski S, Yeung M (2011). Recurrent laryngeal nerve diameter increases during thyroidectomy. Ann Surg Oncol.

[21] Kaya Y, Sarikcioglu L (2015). Sir Herbert Seddon (1903-1977) and his classification scheme for peripheral nerve injury. Childs Nerv Syst.

[22] Serpell JW, Lee JC, Yeung MJ, Grodski S, Johnson W, Bailey M (2014). Differential recurrent laryngeal nerve palsy rates after thyroidectomy. Surgery.

